# Osteolytic effects of tumoral estrogen signaling in an estrogen receptor-positive breast cancer bone metastasis model

**DOI:** 10.20517/2394-4722.2021.27

**Published:** 2021-04-08

**Authors:** Julia N. Cheng, Jennifer B. Frye, Susan A. Whitman, Andrew G. Kunihiro, Julia A. Brickey, Janet L. Funk

**Affiliations:** 1 Cancer Biology Graduate Interdisciplinary Program, University of Arizona, Tucson, AZ 85724, USA.; 2 Department of Medicine, University of Arizona, Tucson, AZ 85724, USA.; 3 Department of Nutritional Sciences, University of Arizona, Tucson, AZ 85724, USA.

**Keywords:** Breast cancer, estrogen receptor, bone metastasis, estradiol, osteolysis, osteoclasts, parathyroid hormone-related protein, bone

## Abstract

**Aim:**

Estrogen receptor α-positive (ER+) subtypes of breast cancer have the greatest predilection for forming osteolytic bone metastases (BMETs). Because tumor-derived factors mediate osteolysis, a possible role for tumoral ERα signaling in driving ER+ BMET osteolysis was queried using an estrogen (E_2_)-dependent ER+ breast cancer BMET model.

**Methods:**

Female athymic Foxn1^nu^ mice were inoculated with human ER+ MCF-7 breast cancer cells via the left cardiac ventricle post-E_2_ pellet placement, and age- and dose-dependent E_2_ effects on osteolytic ER+ BMET progression, as well as direct bone effects of E_2_, were determined.

**Results:**

Osteolytic BMETs, which did not form in the absence of E_2_ supplementation, occurred with the same frequency in young (5-week-old) *vs.* skeletally mature (16-week-old) E_2_ (0.72 mg)-treated mice, but were larger in young mice where anabolic bone effects of E_2_ were greater. However, in mice of a single age and across a range of E_2_ doses, anabolic E_2_ bone effects were constant, while osteolytic ER+ BMET lesion incidence and size increased in an E_2_-dose-dependent fashion. Osteoclasts in ER+ tumor-bearing (but not tumor-naive) mice increased in an E_2_-dose dependent fashion at the bone-tumor interface, while histologic tumor size and proliferation did not vary with E_2_ dose. E_2_-inducible tumoral secretion of the osteolytic factor parathyroid hormone-related protein (PTHrP) was dose-dependent and mediated by ERα, with significantly greater levels of secretion from ER+ BMET-derived tumor cells.

**Conclusion:**

These results suggest that tumoral ERα signaling may contribute to ER+ BMET-associated osteolysis, potentially explaining the greater predilection for ER+ tumors to form clinically-evident osteolytic BMETs.

## INTRODUCTION

Breast cancer is the most common female cancer in the world and the 2nd leading cause of cancer mortality^[1]^. The majority of women with metastatic breast cancer have bone metastases (BMETs), which are primarily osteolytic^[2,3]^. Eighty percent of women with breast cancer BMETs have ER+ tumors due to both the higher incidence of this subtype and its 2-fold greater proclivity to form metastases in bone^[4]^. This association of BMETs in metastatic breast cancer with tumoral ERα expression, which remains highly concordant between primary and bone metastatic tumors^[5–7]^, introduces the possibility that tumor cell ERα signaling within the bone milieu, independent of proliferative effects that are important but not site-specific, may also be driving tumor-associated osteolysis, which is bone-specific, known to be dependent on tumor-derived factors^[8–11]^, and of clear clinical relevance due to the morbidity and mortality associated with osteolytic ER+ BMETs. However, a specific role for ERα signaling in driving tumor-induced osteolysis in ER+ BMET has not, to our knowledge, been previously investigated. Given the frequent association of ERα-positivity with BMETs, this question is highly relevant for the management of breast cancer, particularly since many ER+ BMETs occur post-hormone therapy (HT) and/or are associated with ligand-independent activating ER mutations^[12,13]^. If tumoral E_2_ signaling contributes to ER+ BMET progression by driving tumor-associated osteolysis, targeting of specific downstream signaling pathways mediating this effect could provide novel molecular approaches for skeletal therapeutics to block BMET progression for ER+ tumors.

Because mice, unlike humans, lack aromatase expression in mammary tissue and bone cells^[14,15]^ and also have 10-fold lower circulating 17β-estradiol (E_2_) levels than humans^[16]^, the optimal growth of human ER+ breast cancer orthotopic tumors and osteolytic BMETs in preclinical murine models is dependent on exogenous E_2_ supplementation^[17,18,19–26,27,28]^. This presents a challenge when studying murine models of human ER+ BMETs given the responsiveness of both tumor and bone cells to E_2_^[29–33]^ and the absence of syngeneic models of murine ER+ breast cancer BMET. Indeed, the E_2_ doses required to promote ER+ breast cancer growth in osteolytic xenograft models also increase murine bone mass^[18,20,21,26,28,34]^ and furthermore, can induce osteolytic murine osteosarcomas in some animals, as previously demonstrated by our laboratory^[34]^.

Evidence from ER-BMET models, which represent the majority of preclinical breast cancer BMET research, has allowed for an assessment of the influence of estrogenic effects on the bone microenvironment, independent of tumor cell ER signaling, on osteolytic ER− BMET progression. Taken together, these ER− breast cancer xenograft studies suggest that both induction of bone formation by E_2_-treatment^[35,36]^ and bone resorption by E_2_-deprivation [via ovariectomy (OVX)]^[28,37,38]^ promote ER− BMET progression. In ER+ BMET models, E_2_ bone-microenvironmental effects have often not been considered^[19,21–26,39,40]^ and are rarely documented^[18,20,28]^, while a role of tumor ERα signaling in driving tumor-associated osteolysis has not, to our knowledge, previously been studied.

To address these knowledge gaps regarding the effects of ERα signaling in the tumor itself *vs.* the bone microenvironment in driving tumor-associated osteolysis and osteolytic progression for ER+ BMET, E_2_ effects were assessed in a murine model of osteolytic ER+ BMET using intracardiac (IC) injection of ER+ human breast cancer cells. Studies included an exploration of dose- and age-dependent effects of E_2_ with the goal of identifying conditions under which E_2_ effects on bone turnover could be accounted for separately from direct effects of E_2_ on ER+ tumor-mediated osteolysis. The primary objective of these studies was to determine whether tumoral ERα signaling, in addition to well-known proliferative effects that are not site-specific, could be driving osteolysis within the bone microenvironment, thus potentially explaining the greater proclivity of ER+ (*vs.* ER−) breast cancer cells to form clinically evident osteolytic BMET.

## METHODS

### Cell lines and culture

Human ER+ breast cancer tumor cell lines, MCF-7, T47D, and ZR-75-1 [American Type Culture Collection (ATCC), Manassas, VA], or bone-tropic ER− human MDA-MB-231 (MDA-SA) cells^[41,42]^, generously provided by Dr. Theresa Guise, were cultured in E_2_-replete Dulbecco’s modified Eagle’s medium (DMEM; Invitrogen, Carlsbad, CA) or RPMI-1640 (Invitrogen), as per ATCC’s recommendation, containing 10% of heat-inactivated fetal bovine serum (FBS; Atlanta Biologicals, Flowery Branch, GA), 1% of penicillin/streptomycin (Thermo Fisher, Waltham, MA) in 37 °C, and 5% of CO_2_ in a humidified atmosphere. All human cell lines were authenticated, as previously described^[41,43]^, including MCF-7 BMET-derived tumor cells used in parathyroid hormone-related protein (PTHrP) secretion experiments, which were isolated from osteolytic BMET-bearing limbs 42–56 days post-inoculation and passaged twice to remove non-immortalized and non-adherent murine cells prior to authentication.

### Animal studies

All animal protocols were approved by the Institutional Animal Care and Use Committee at The University of Arizona (UA) in accordance with the National Institutes of Health Guide for the Care and Use of Laboratory Animals. Four or 15-week-old female *Foxn1*^*nu*^ athymic nude outbred mice (Envigo, Indianapolis, IN) were received and housed in plastic cages (maximum 5/cage) in laminar flow isolated hoods with ad libitum access to water and autoclaved mouse chow (NIH-31 Modified diet, Envigo). The number of animals required was determined a priori with the statistical goal of detecting a significant difference of osteolytic lesion area between groups assuming a moderate effect size with α = 0.05 and β = 0.80 (G*Power Software v3.1)^[44]^. Mice (*n* = 8–13/group) were inoculated at approximately 5- or 16-week of age with 1 × 10^5^ human breast cancer cells (MCF-7, MDA-SA, T47D, or ZR-75-1) via the left cardiac ventricle (IC), as previously described^[41]^, either in estrogen-naive mice, or in estrogen-supplemented mice 3 days post-placement of 60-day extended-release 17β-estradiol (E_2_) pellets (0.05, 0.10, 0.18, 0.36, or 0.72 mg, Innovative Research of America, Sarasota, FL)^[34]^. In separate experiments, as indicated, mice *not* inoculated with tumor cells (tumor-naive) were similarly treated with E_2_ pellets to determine effects on bone turnover independent of tumor-associated osteolysis. Additionally, to examine the possible influence of E_2_ supplementation on tumor cell dissemination to bone, mice 3-days post-E_2_ pelleting (*vs.* controls, *n* = 3–5/group) were inoculated with 8 × 10^5^ MCF-7 cells freshly labeled with Vybrant DiD, as per the manufacturer’s instructions (Thermo Fisher), with fluorescent membrane staining remaining detectable for up to 7 days in culture. Twenty-four hours post-inoculation, cells were isolated from each proximal (25%) tibia, the most common and earliest site of BMET (data not shown), by flushing with media and repeated washing and crushing of bone. Cells thus isolated were seeded into 48-well cell-culture plates (3 wells/tibia) and allowed to adhere overnight prior to imaging of DiD-positive cells using the Cy5 filter of a Keyence BZ-X700 fluorescent microscope (Keyence Corporation of America, Itasca, IL). DiD-positive (DiD+) tumor cells, quantified using ImageJ software (National Institutes of Health, NIH), are reported as DiD+ cells/10^6^ bone marrow cells for each tibia. Similarly isolated cells from tumor-naive mice were included as negative controls, and cultured MCF-7 cells 24 h-post DiD labeling were used as positive microscopy controls. No changes in health status necessitating euthanasia occurred in mice, which were also examined 6-week post-tumor cell inoculation at gross necropsy for non-bone metastases, as determined by researchers and UA veterinary staff: rare unanticipated deaths were attributable to anesthesia, weights were unchanged in E_2_-treated and/or tumor-bearing mice *vs.* controls, and urinary retention, a well-characterized side-effect of E_2_-supplementation in mice^[45,46]^ that was not severe enough to warrant early termination, was observed in 20%–30% of mice treated with higher E_2_ doses, as previously reported^[34]^.

### Radiographic determination of osteolytic BMET lesions and E_2_ effects on bone

To assess the size and incidence of radiographically-evident osteolytic BMET lesions, radiographs of mouse hind limbs (Faxitron UltraFocus 1000, Faxitron Bioptics, Tucson, AZ) in E_2_-supplemented tumor cell-inoculated mice were obtained weekly over the 6-week course of experiments. Radiographic osteolytic lesion incidence and area per mouse were determined in a blinded fashion with radiographic images assessed by three independent investigators using ImageJ software (NIH)^[34]^. Because E_2_ can induce osteolytic osteosarcomas in nude mice^[34]^, the identity of osteolytic BMETs in E_2_-supplemented mice was verified by correlating radiographic lesions in each hind limb bone with histologic evidence of cytokeratin-positive breast cancer tumors^[20,34]^ prior to calculating radiographic osteolytic BMET incidence or lesion area per mouse. When calculating average osteolytic lesion size, mice lacking osteolytic BMET were excluded so that E_2_ effects on lesion size could be assessed independent of effects on incidence. In parallel experiments to determine E_2_ effects on bone in the absence of osteolytic BMET, dual-energy X-ray absorptiometry (DXA) was performed weekly in E_2_-supplemented mice not inoculated with tumor cells (tumor naive) to assess changes in tibial areal bone mineral density (aBMD) (Faxitron UltraFocus 1000)^[34]^. At termination of these 6-week experiments, to examine E_2_ effects specific to trabecular bone, microcomputed tomography (microCT) imaging was performed *ex vivo* in a subset of mice to assess proximal tibial metaphyseal trabecular bone volume/total volume (BV/TV) by the Endocrine Research Unit at the San Francisco VA Medical Center (Scanco microCT 50, Scanco Medical, Basserdorf, Switzerland) as previously described^[34,47]^.

### Bone metastatic breast cancer tumor histology and bone histomorphometry

Hind limbs were removed either 2 weeks (bone histomorphometry) or 6 weeks (bone histomorphometry or immunohistochemical analysis of Ki67, ER, or cytokeratin) post-tumor cell inoculation, fixed, decalcified, and paraffin-embedded for histologic analyses of midsagittal (approximate depth of 400–500 μM) 5–6 μM sections as previously described^[48]^. For measuring histologic breast cancer tumor size (tumor burden), epithelial MCF-7 breast cancer tumors were identified using a pan-cytokeratin primary antibody (#Z0622, Agilent Dako, Santa Clara, CA) and continued expression of ERα was verified using a human ERα primary antibody (#ab108398, Abcam, Cambridge, United Kingdom) using previously described immunohistochemical (IHC) methods^[48]^. Cytokeratin-positive breast cancer tumor area in hind limbs bones was determined in a blinded fashion (expressed per leg as % of tumor area/bone area). Proliferating breast cancer cells in bone were identified using a primary antibody directed against a human Ki67 proliferation marker (#D2H10, Cell Signaling, Danvers, MA). Breast cancer tumor cell proliferation in each hind limb was assessed in a blinded fashion by calculating the average number of Ki67-positive tumor cells in four high power fields per bone (expressed per hind limb as % of total tumor cells), with the mean for each treatment group determined by averaging values for each limb. In addition, osteoblasts-identified as hematoxylin-stained mononuclear cuboidal cells lining the bone surface-or multinucleated tartrate-resistant acid phosphatase (TRAP)-positive osteoclasts were quantified in the tibial metaphyses of tumor-naive mice or at the bone tumor interface of tumor-bearing mice, as per ASBMR nomenclature Committee Guidelines and using standard methods, as previously described^[34,41,49–53]^. Osteoclasts or osteoblasts in tumor-naive mice are reported as cell number per mm of bone surface (BS) or per tissue area (mm^2^), and osteoclasts in tumor-bearing hind limbs of mice are reported as cell number per mm of bone at the tumor-bone interface^[34,41,49,54,55]^. All images for immunohistology and bone histomorphometry were analyzed using ImageJ software (NIH).

### Serum markers of bone turnover or estradiol

Serum markers of bone formation [rat/mouse P1NP EIA; Immunodiagnostic Systems (IDS), United Kingdom] or bone resorption (mouse CTX-1; IDS) were measured in fasting serum collected 2 weeks after the start of E_2_ supplementation (*vs.* age-matched controls) using commercially available ELISA kits^[18,28]^ as previously described^[34]^. E_2_ levels in serum collected 2 weeks post pellet placement were assayed by the University of Virginia Center for Research in Reproduction Ligand Assay and Analysis Core using a commercially available 17β-estradiol ELISA developed for use in mice (Calbiotech, El Cajon, CA)^[56]^. All sera were stored at −80 °C prior to assay.

### PTHrP assay

To analyze PTHrP secretion from ER+ tumor cells and its E_2_ dependency, ER+ MCF-7 cells or ER+ tumor cells isolated from MCF-7 BMET were plated in 24-well plates at a density of 1.3 × 10^5^ cells/well in E_2_-depleted media [phenol red-free DMEM (Invitrogen), 10% charcoal-stripped FBS (Valley Biomedical, Winchester, VA), 1% penicillin/streptomycin (Thermo Fisher), and 200 mM L-Glutamine (Sigma Aldrich, St. Louis, MO)] for 4 days, during which time cell number did not change for any cell line (data not shown), prior to treatment with E_2_ (10^−11^-10^−6^ M, as indicated; Sigma Aldrich), an ERα specific agonist propyl pyrazole triol (PPT; 10^−8^ M; Tocris, Minneapolis, MN), an ERα specific antagonist methyl-piperidinopyrazole hydrate (MPP; 10^−6^ M, Tocris), or vehicle control for 48 or 52 h, as indicated. Conditioned media, stored at −80 °C after addition of protease inhibitors (Sigma Aldrich), were assayed for secreted PTHrP using a commercial immunoradiometric assay (Beckman Coulter, Brea, CA). A lack of treatment effect on cell number during the 48 or 52-h incubation was verified using a commercial MTT assay (ATCC).

### Statistical analyses

Unless otherwise noted, data are reported as mean ± SEM, with statistical significance of 2-sided *P*-values defined as *P* ≤ 0.05. Statistical differences were determined using Prism 8.0 software (Graphpad, San Diego, CA) for 1- or 2-way analyses of variance (ANOVA) with *post-hoc* testing as well as tests for log-rank, mixed-effects, and *t*-test, as indicated. Analyses of skeletal parameters in tumor naive mice were not corrected for multiple comparisons, using Fisher’s LSD test, to maximize the possibility of detecting dose-dependent E_2_ effects (although none were found)^[57]^.

## RESULTS

### E_2_-dependent osteolytic ER+ MCF-7 BMET progression in young vs. skeletally mature E_2_ (0.72 mg)-supplemented mice

**Figure 1.**
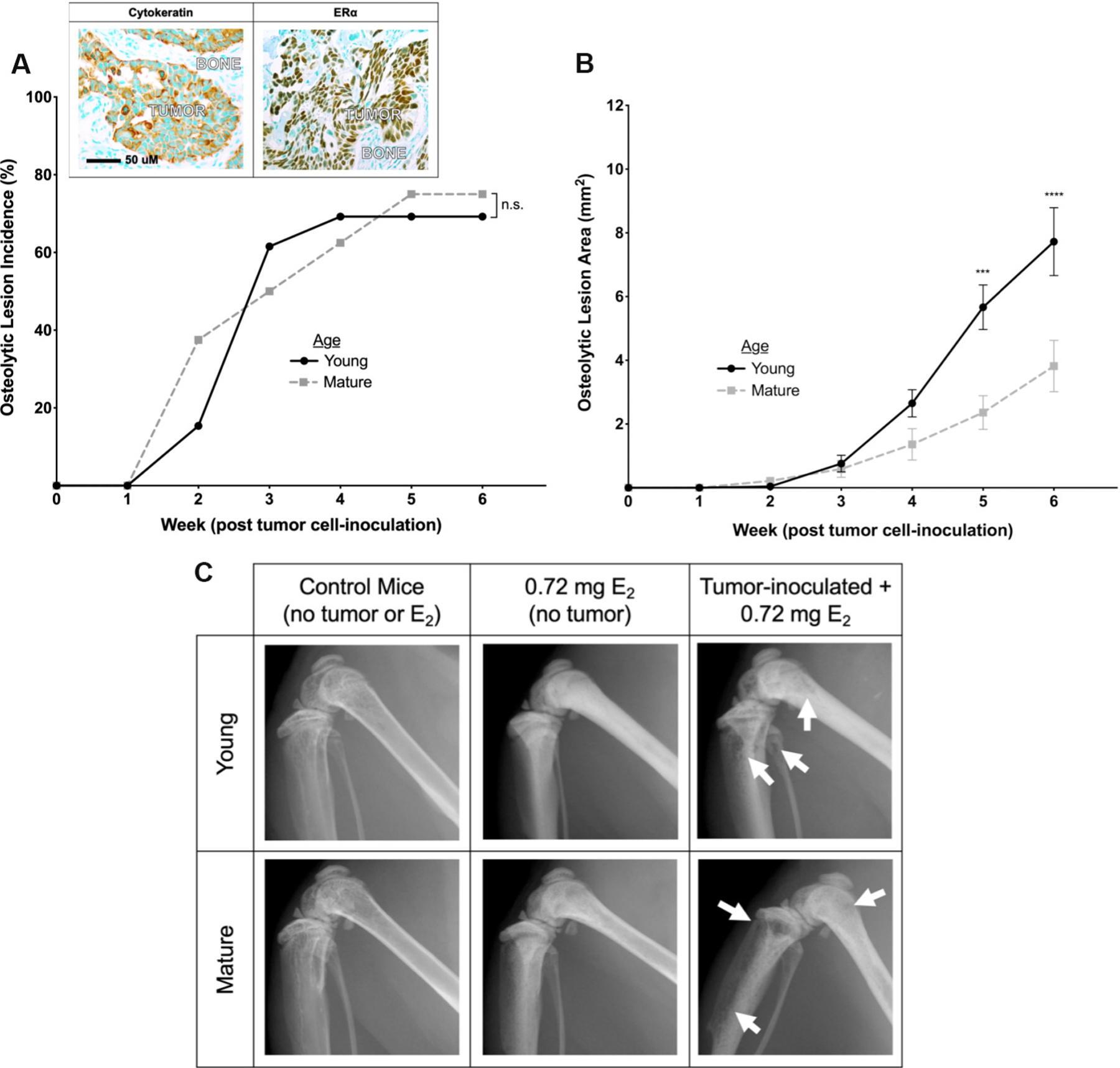
Comparison of osteolytic ER+ BMET progression in young *vs.* skeletally mature mice supplemented with 0.72 mg E_2_. (A) Osteolytic lesion incidence and (B) osteolytic lesion area in young (5-week-old) and skeletally mature (16-week-old) mice supplemented with 0.72 mg E_2_ and inoculated with ER+ tumor cells (*n* = 8–13/group). Inset, representative immunohistochemical (IHC) images demonstrating cytokeratin+ (left panel; brown), ERα+ (right panel; brown) human breast cancer tumors in tibiae. ***P ≤ 0.001, ****P ≤ 0.0001 young *vs.* skeletally mature mice, by 2-way ANOVA with Sidak’s post-test. There was no significant difference (n.s.) in osteolytic lesion incidence by Log-rank (Mantel-Cox) test. (C) Representative hind limb radiographs in young (top) *vs.* mature (bottom) age-matched control (left panels), naive E_2_ (0.72 mg)-supplemented (middle panels), or tumor cell-inoculated and E_2_ (0.72 mg)-supplemented mice (right panels) 6 weeks post-inoculation and E_2_ supplementation. Osteolytic lesions are marked by arrows.

Osteolytic BMETs were not detected in the absence of E_2_ supplementation when young (5-week-old) mice inoculated with MCF-7 cells were followed for up to 8 months (data not shown). When supplemented with an E_2_ dose (0.72 mg) supporting *in vivo* MCF-7 orthotopic tumor growth^[17,58]^, radiographic osteolytic breast cancer lesions were evident as early as 2 weeks post-MCF-7 tumor cell inoculation of young (5-week old) mice (Figure 1A and inset), reaching a maximal incidence of 69% within 4 weeks with continuous size increases over the 6-week course of the experiment [Figure 1B], without evidence of metastases at non-bone sites. BMET formation in E_2_ (0.72 mg)-supplemented MCF-7-inoculated mice contrasted with results in 5-week-old mice inoculated with T47D or ZR-75-1 cells, where no osteolytic BMETs were noted (data not shown). When skeletally mature (16-week-old) mice supplemented with the same 0.72 mg E_2_ dose were inoculated with MCF-7 cells, the progression time course and incidence of osteolytic BMET lesion formation were the same as those in 5-week mice [Figure 1A]; however, osteolytic lesion size was significantly smaller [Figure 1B]. Radiographs documenting proximal tibial and distal femoral osteolytic lesions, also common sites for ER− BMETs^[59]^, were also notable for clear evidence of E_2_-driven, albeit possibly differential, increases in bone density in mice of both ages [Figure 1C]. This observation raised questions about possible contributions of E_2_ effects on the bone microenvironment (*vs.* direct effects on ER+ tumors) in supporting ER+ MCF-7 BMET progression, a postulate further supported by findings in 5-week-old mice inoculated instead with osteotropic ER− MDA-MB-231 cells, where treatment with the same 0.72 mg E_2_ dose led to an increase in osteolytic lesion size (3.5 ± 0.8-fold increased lesion size in E_2_-supplemented (*n* = 12) *vs.* control mice (*n* = 10), *P* < 0.01), but unchanged incidence (91.6% *vs.* 80.0%, *P* > 0.05), consistent with prior reports of pro-metastatic, anabolic E_2_ bone effects in ER− BMET models^[35,36]^.

### Differential effects of E_2_ (0.72 mg) on bone turnover in tumor-naive young vs. skeletally mature mice

**Figure 2.**
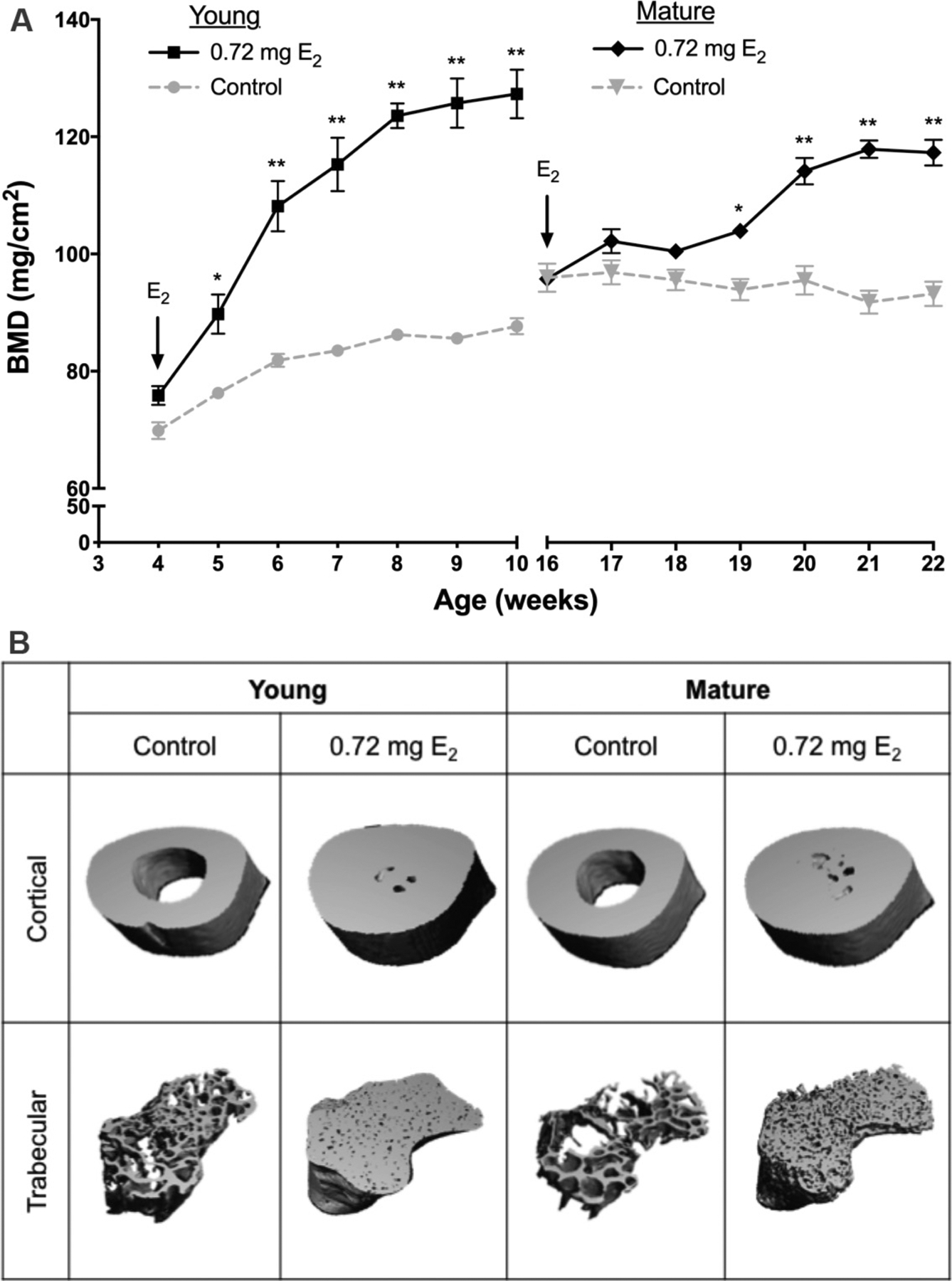
Effects of 0.72 mg E_2_ on bone mineral density and structure in tumor-naive young *vs.* skeletally mature mice. (A) Tibial areal bone mineral density (BMD) in young (4-week-old) *vs.* skeletally mature (15-week-old) mice supplemented with 0.72 mg E_2_ (*vs.* control), as measured by DXA (*n* = 6–8/group). Arrows indicate time of E_2_ pellet placement. *P ≤ 0.01, **P ≤ 0.0001 *vs.* age-matched control, by 2-way ANOVA with Sidak post-test. (B) Representative microCT images of tibial cortical (top) and trabecular (bottom) bone of young *vs.* skeletally mature E_2_ (0.72 mg)-supplemented mice (*vs.* age-matched controls), 6 weeks after E_2_ pellet placement.

Because radiographs suggested that anabolic effects of E_2_ (0.72 mg) on bones in tumor-bearing mice could be age-dependent, direct bone effects of this E_2_ dose (0.72 mg) were quantified in tumor-naive mice of both ages to assess the postulate that E_2_ (0.72 mg)-driven ER+ BMET osteolytic lesion size was greater in mice whose bones yielded a greater anabolic E_2_ response (i.e., young mice). Total tibial aBMD increased significantly in response to E_2_ (0.72) over 6 weeks of supplementation in tumor-naive mice of both ages [Figure 2A], but with a larger increase in younger mice (68% *vs.* 23%), whose BMD was lower at baseline and still increasing in untreated age-matched controls. Cross-sectional microCT images [Figure 2B] confirmed dramatic, but differential, effects of E_2_ on both cortical and trabecular bone in the tibiae of skeletally young *vs.* mature mice after 6 weeks of treatment. In the proximal tibial, a frequent osteolytic BMET site, aBMD and trabecular BV/TV increased significantly in response to E_2_ in mice of both ages [Table 1]; however, the increase was significantly greater in young mice [e.g., BV/TV of 91% *vs.* 60% (*P* < 0.0001) in young *vs.* mature, respectively].

In skeletally mature mice, P1NP, a serum marker of bone formation, was significantly lower (*vs.* young mice) and unchanged by 0.72 mg E_2_-supplementation, contrasting with a 0.72 mg E_2_-induced increase in already significantly higher P1NP levels in young mice, such that P1NP levels were 3.1-fold higher (*P* < 0.0001) in E_2_-treated young *vs.* skeletally mature mice [Table 1]. Osteoblast number (N.Ob) per bone surface tended to increase in response to E_2_ treatment in mice of both ages; however, these trends were not statistically significant. Given the large increase in bone surface, N.Ob per area was also calculated and was only significantly increased in response to E_2_ in young mice, resulting in Ob counts that were 1.6-fold higher in young (*vs.* mature) E_2_-treated mice (*P* < 0.05), consistent with higher circulating P1NP levels [Table 1].

Age-related differences in bone resorption in E_2_-treated mice were less evident in these ovary-intact mice. While osteoclast number (N.Oc) per bone surface was not changed by E_2_ treatment in mice of either age, N.Oc per bone area increased significantly and similarly in age of both ages, such that there was no difference in Oc counts in young *vs.* mature E_2_ treated mice [Table 1]. Similarly, CTX-1 levels in 0.72 mg E_2_-treated mice of both ages were the same [Table 1]. *In toto*, these data demonstrate that the greater net increases in bone in young (*vs.* mature) mice treated with same E_2_ dose were attributable to higher rates of bone formation, which were positively associated with osteolytic lesion size, but not incidence, in tumor-inoculated young (*vs.* mature) mice supplemented with the same E_2_ (0.72 mg) dose.

### Assessing dose-dependency of E_2_ effects on bone turnover in tumor-naive *vs.* progression of osteolytic ER+ BMET lesions in ER+ tumor cell-inoculated 5-week-old mice

**Figure 3.**
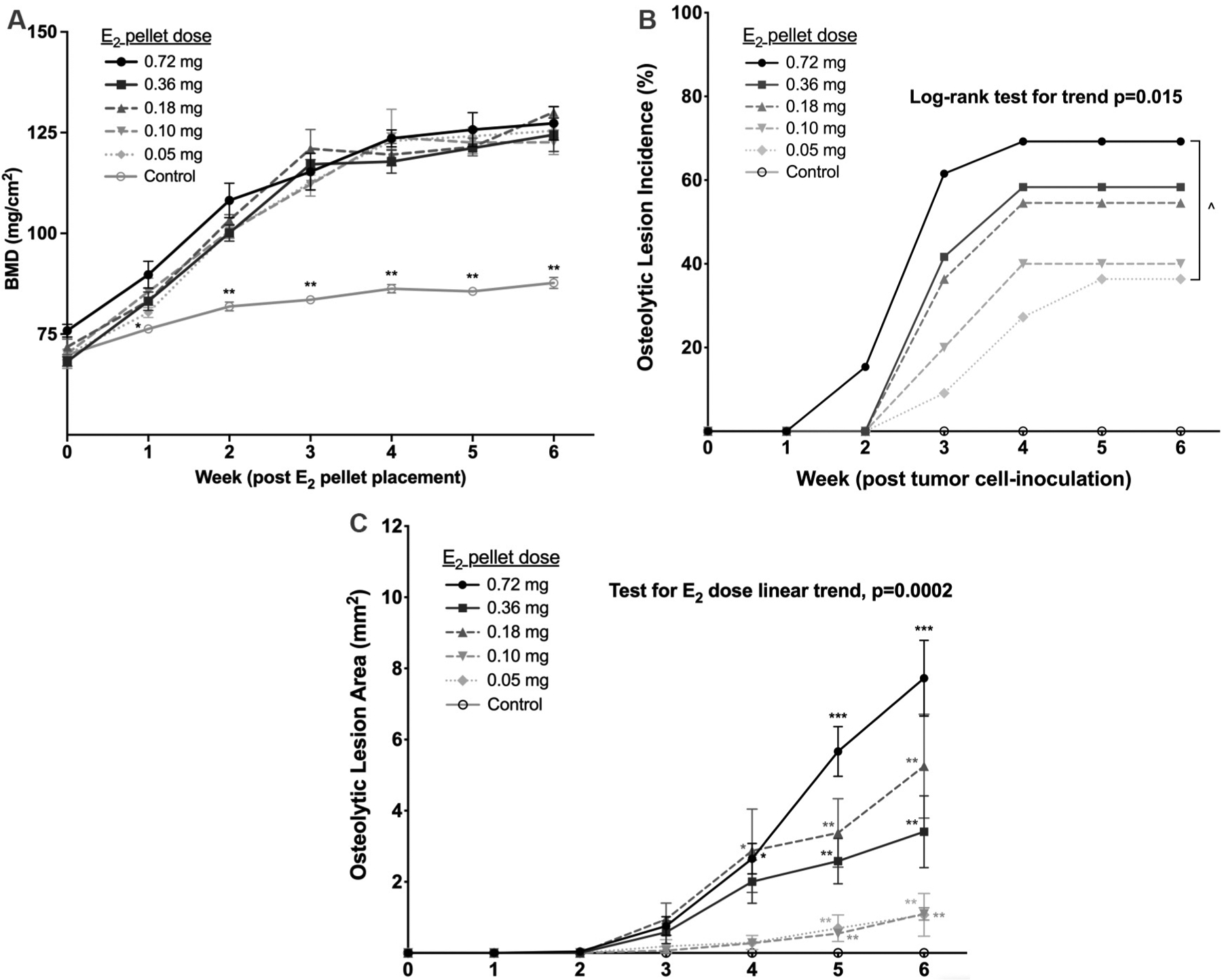
Comparison of dose effects of E_2_ on bone mineral density in tumor-naive *vs.* osteolytic ER+ BMET progression in tumor-inoculated mice. (A) Areal bone mineral density (BMD) of tibiae in tumor-naive 4-week-old mice treated with the indicated doses of E_2_ (*vs.* age-matched controls), as measured by DXA (*n* = 6–8/group). *P ≤ 0.01 0.72 mg E_2_ *vs.* control; **P ≤ 0.01 for each E_2_ dose *vs.* control, with no statistical differences between E_2_ doses, by mixed-effects analysis with Tukey post-test. (B) Osteolytic lesion incidence and (C) osteolytic lesion area in age-matched mice inoculated at 5 weeks of age with ER+ tumor cells (*n* = 10–13/group) 3 days post-supplementation with the same E_2_ doses (*vs.* no E_2_ controls; open circles). *P*-values for E_2_ dosing trends were calculated using Kaplan Meier analysis with log-rank test for incidence, or 1-way ANOVA of AUC data with post-test for linear trend for lesion area. ^*P* ≤ 0.05 0.72 mg E_2_ *vs.* 0.05 mg E_2_ by log-rank (Mantel-Cox) test. *P ≤ 0.05 *vs.* controls or 0.05 mg E_2_; **P ≤ 0.05 *vs.* control, 0.05, 0.10, or 0.72 mg E_2_; ***P ≤ 0.05 *vs.* every dose, by 2-way ANOVA with Holm-Sidak post-test.

Because significant E_2_ effects on bone occurred in mice of both ages, bone effects of a range of lower E_2_ doses previously reported to support dose-dependent growth of orthotopic MCF-7 tumors *in vivo*^[17]^ were next assessed in mice of a single age to determine whether an E_2_ dose could be identified that did not significantly alter bone. Remarkably, E_2_-induced increases in total tibial aBMD were identical for all doses, plateauing 3 weeks after E_2_-pellet placement in 5-week-old mice [Figure 3A]. Other bone parameters including proximal tibial aBMD or BV/TV, bone turnover markers, and N.Ob or N.Oc were also similarly increased in response to the lowest E_2_ dose (0.05 mg) *vs.* the highest E_2_ dose (0.72 mg) tested, without any dose-dependence [Table 1]. Having documented essentially identical bone microenvironment effects over this entire range of E_2_ doses known to support *in vivo* MCF-7 proliferation at orthotopic sites, with evidence of dose-dependent increases in circulating E_2_ levels across the range of doses [Supplementary Figure 1], the effects of this E_2_ dosing regimen on ER+ BMET progression in MCF-7 cell-inoculated mice of the same age were assessed. In mice treated with increasing doses of E_2_, osteolytic ER+ BMET lesions formed in a dose-dependent fashion with respect to both incidence and size (Figure 3B–C, *P* ≤ 0.05), with the highest dose of E_2_ tested (0.72 mg) forming osteolytic lesions with a similar size and incidence 6 weeks post-tumor inoculation as occurs in a commonly used non-E_2_-supplemented ER− MDA-MB-231 BMET model at week 3 ^[41]^. The 7-fold larger size and higher incidence of osteolytic lesions in MCF-7-inoculated mice treated with the highest (0.72 mg) *vs.* lowest (0.05 mg) E_2_ dose contrasts with dose-dependent effects of E_2_ in mice inoculated instead with ER− MDA-MB-231 cells; ER− osteolytic lesion size, while increased by E_2_ treatment, as described above, was not statistically different in mice treated with the highest (0.72 mg) *vs.* lowest (0.05 mg) E_2_ doses (*P* = 0.11, *n* = 9–12) and osteolytic lesion incidence was unchanged by any E_2_ dose (*vs.* non-supplemented controls, data not shown). Thus, while bone anabolism and osteolytic ER− BMET lesion progression were each induced by E_2_ in 5-week-old mice, neither exhibited E_2_-dose dependence; in contrast, the size and incidence of osteolytic ER+ BMET lesion progression were E_2_ inducible and E_2_-dose dependent.

### Assessing possible E_2_ dose-dependency of ER+ tumor cell dissemination to bone

Because E_2_ pellets were placed 3 days prior to tumor cell inoculation to allow for stabilization, studies were undertaken to assess possible dose-dependent E_2_ effects on ER+ tumor cell dissemination to bone. Following inoculation of DiD-labelled MCF-7 cells, DiD+ tumor cells detected in the proximal tibia - while trending slightly higher in E_2_-treated *vs.* control mice 24 h post-inoculation [Supplementary Figure 2] - were not statistically different. Most importantly, there was no evidence of a dose-dependent E_2_ effect in mice treated with the lowest (0.05 mg) *vs.* highest (0.72 mg) E_2_ doses tested [Supplementary Figure 2]. There was also no evidence of an age effect, when comparing bone disseminated DiD+ MCF-7 cells in young *vs.* skeletally mature mice treated with 0.72 mg E_2_ [Supplementary Figure 2].

### Assessing possible E_2_ dose-dependency of ER+ tumor burden and proliferation in bone

**Figure 4.**
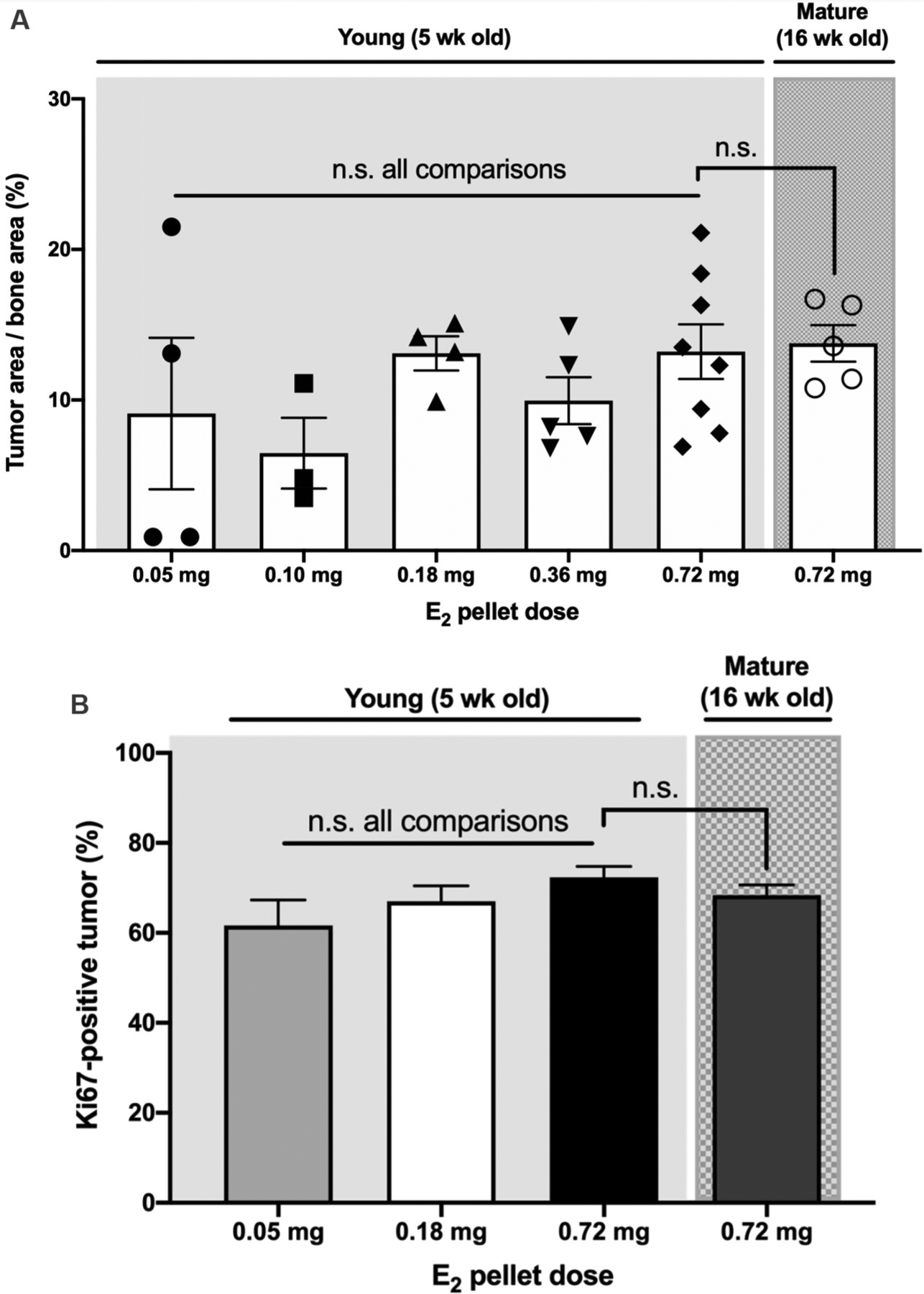
E_2_ effects on histologic tumor burden and tumor cell proliferation in bone. (A) Cytokeratin-positive breast cancer tumor area in hind limbs, normalized to bone area in mid-sagittal sections, 6 weeks post-ER+ tumor cell inoculation of 5- or 16-week old mice. There was no linear trend in tumor burden with increasing E_2_ doses, and no significant differences (n.s.) between E_2_ doses, or between young and mature mice treated with 0.72 mg E_2_, as tested by 1-way ANOVA with Sidak post-test (*n* = 3–9/group). (B) Proliferating, Ki67-positive cells in hind limb breast cancer tumors (% of total) 6 weeks post tumor-inoculation. There were no significant differences (n.s) in the proportion of Ki67-positive tumor cells between E_2_ doses [including the lowest (0.05 mg) and highest (0.72 mg)], or between young (5-week) and mature (16-week) mice treated with 0.72 mg E_2_, as calculated by 1-way ANOVA with Sidak post-test (*n* = 8–18/group).

Because proliferative effects of E_2_ on ER+ MCF-7 cells are well described *in vitro* and *in vivo* at orthotopic sites^[17,58]^, a possible E_2_ dose-dependency for histologic tumor burden (size) and tumor cell proliferation in bone were assessed 6 weeks post-inoculation, when osteolytic lesion size was still increasing. While the mean area for cytokeratin+ ER+ breast cancer tumors in bone tended to be smaller for lower E_2_ doses, the range of tumor sizes was similar across doses without a statistical difference in mean values [Figure 4A]; nor was there a significant linear trend for increasing doses. Tumor burden in 0.72 mg E_2_-pelleted young *vs.* skeletally mature mice was also not statistically different [Figure 4A]. Tumor cell proliferation, assessed by Ki67-positivity, was notable in E_2_-supplemented mice (> 60%), but again was without E_2_-dose or age-dependency [Figure 4B].

### Assessing E_2_ dose-dependency of ER+ tumor-associated osteolysis

**Figure 5.**
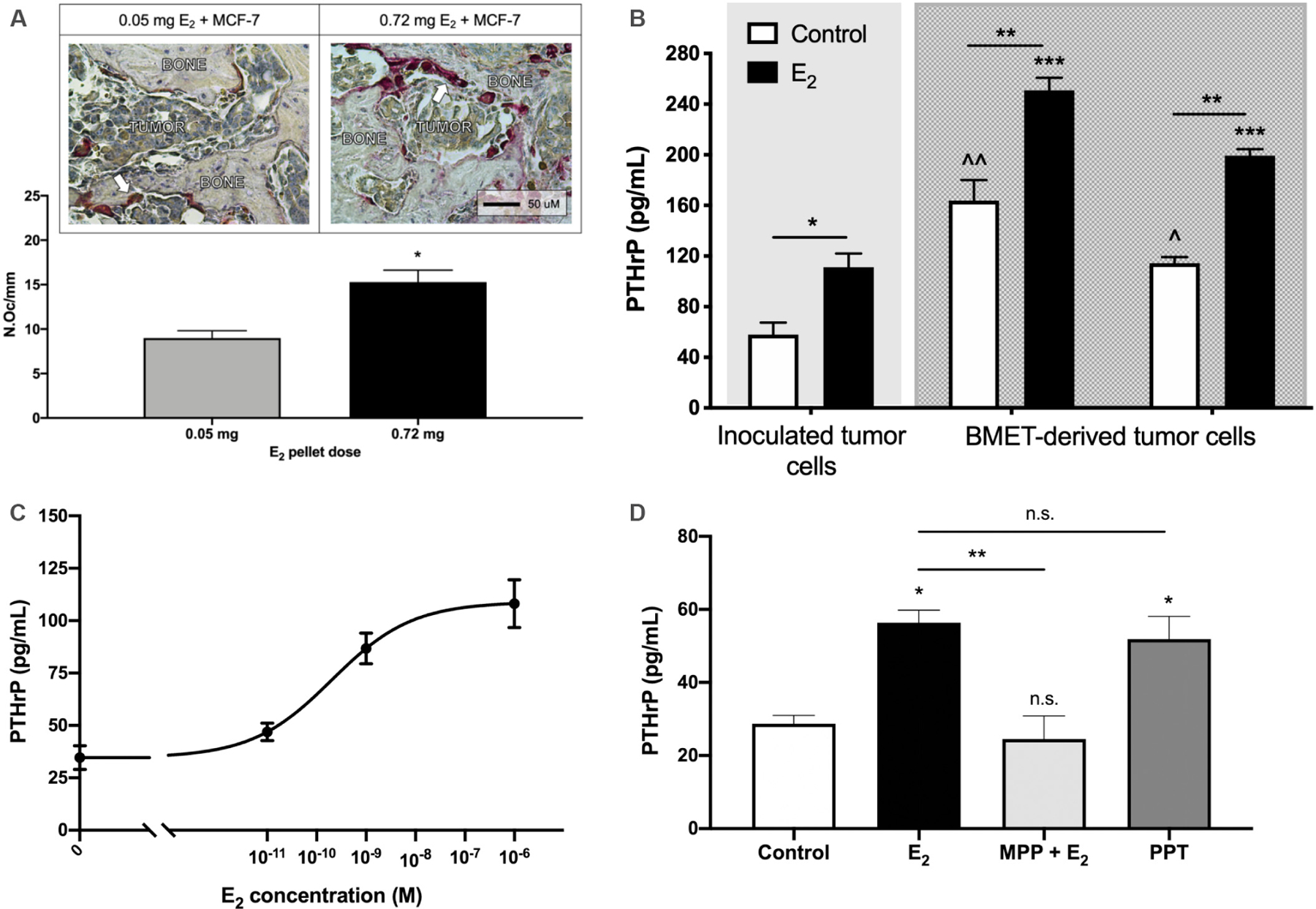
E_2_ effects on tumoral osteolysis and secretion of osteolytic PTHrP. (A) Osteoclast number at tumor-bone interface (N.Oc/mm) in tibiae from tumor cell-inoculated mice supplemented with the lowest (0.05 mg) and highest (0.72 mg) E_2_ doses (*n* = 8–11 tibiae/group), with representative images of TRAP-positive multinucleated osteoclasts (see arrow for example of red-stained multinucleated osteoclast). *P < 0.01 by *t*-test. (B) Osteolytic PTHrP secretion from inoculated tumor cells *vs.* ER+ tumor cells isolated from BMETs of 2 different mice. Cells were treated with 10^−7^ M E_2_ or media control for 48 h after 4 days in E_2_-free media (*n* = 4/group). Cell number, as assessed by MTT assay, was not different between cell lines or altered by E_2_ treatment (data not shown). *P ≤ 0.05, **P ≤ 0.001 E_2_ *vs.* control; ^*P* ≤ 0.05, ^^*P* ≤ 0.0001 inoculated *vs.* BMET-derived control cells; ***P ≤ 0.001 inoculated *vs.* BMET-derived E_2_-treated cells, by 2-way ANOVA with Tukey post-test. (C) E_2_ dose-dependency of PTHrP secretion in MCF-7 maintained in E_2_-deplete media for 4 days prior to treatment with various concentrations of E_2_ (M), as indicated, for 72 h (*n* = 3–4/group). (D) PTHrP secretion from ER+ BMET-derived cells treated with E_2_ (10^−8^ M), E_2_ and MPP (10^−6^ M, ERα antagonist), PPT (10^−8^ M, ERα agonist), or media control for 52 h after 4 days in E_2_-deplete media (*n* = 3–4/group). Cell numbers (by MTT assay) were not altered by treatments (data not shown). *P ≤ 0.05 *vs.* control; **P ≤ 0.01 E_2_ *vs.* MPP + E_2_; not significant (n.s.) *vs.* control or as shown, by 1-way ANOVA with Sidak post-test. MPP: Methyl-piperidino-pyrazole; PPT: propyl pyrazole triol; PTHrP: parathyroid hormone-related protein.

Having eliminated differential tumor cell dissemination or proliferative effects of E_2_ as being responsible for the E_2_ dose-dependence of ER+ osteolytic BMET lesion progression, dose-dependent effects of E_2_ on tumor-associated osteolysis-specific mechanisms were next assessed. While E_2_ suppresses osteoclast numbers in estrogen-deficient bone^[60]^, in ovary-intact tumor-naive mice, neither the highest (0.72 mg) nor the lowest (0.05 mg) E_2_ dose altered osteoclast numbers per bone surface at 2 weeks [Table 1] or 6 weeks (data not shown). However, consistent with E_2_ dose-dependent increases in ER+ BMETs osteolytic lesion size and incidence [Figure 3B–C], the number of bone-resorbing osteoclasts at the tumor-bone interface of ER+ tumor cell-inoculated mice treated with the highest (0.72 mg) E_2_ dose was significantly greater than that in mice treated with the lowest (0.05 mg) E_2_ dose, where osteoclast numbers on bone surfaces interfacing with tumors [Figure 5A] were not different from those in age-matched, tumor-naive control mice [N.Oc/BS, 10.9 ± 1.8 (*n* = 6), *P* > 0.05]. The osteolytic factor, parathyroid hormone-related protein (PTHrP), which is expressed in most clinical breast cancer BMET^[8,11,59,61–63]^, was secreted constitutively from ER+ MCF-7 tumor cells used for inoculation, while constitutive PTHrP secretion from ER+ tumor cells isolated from MCF-7 BMET lesions was 2- to 3-fold higher (*P* ≤ 0.05) [Figure 5B]. In both inoculated and BMET-derived cells, tumoral PTHrP secretion was further increased (*P* ≤ 0.05) in response to E_2_ treatment, resulting in 2-fold higher levels of E_2_-induced PTHrP secretion from BMET-derived (*vs.* inoculated) ER+ tumor cells. As with *in vivo* BMET-associated osteolysis, E_2_-inducible PTHrP secretion *in vitro* was also dose-dependent [Figure 5C]. Moreover, E_2_ induction of PTHrP secretion was ERα-mediated; MPP, an ERα-specific antagonist^[64]^ that did not alter tumoral PTHrP secretion (data not shown), blocked E_2_-induced PTHrP in BMET-derived tumor cells (Figure 5D; *P* ≤ 0.01). Furthermore, PPT, an ERα specific agonist with an affinity for ERα similar to that of E_2_ (and 410-fold higher for ERα *vs.* ERβ)^[65]^, significantly induced PTHrP to identical levels as compared to an equimolar concentration of E_2_ [Figure 5D].

## DISCUSSION

Anti-estrogen hormone therapies and bisphosphonates each have a proven benefit in reducing the development and progression of osteolytic ER+ BMETs; however, BMETs still occur in ~80% of women with ER+ metastatic breast cancer and remain incurable^[4,66–71]^. The recent addition of agents acting downstream of ERα to decrease proliferation (CDK4/6 inhibitors), while not curative, has yielded significant benefits^[72]^, likely due in part to the high prevalence of ligand-independent, activating ERα mutations in ER+ metastatic breast cancer^[12]^. Similarly, if separate osteolytic effects of tumoral ERα signaling are also demonstrated to drive ER+ BMET progression, novel molecular approaches targeting specific tumoral osteolytic pathways downstream of ERα could provide new avenues for skeletal therapeutics to block BMET progression for ER+ tumors, which comprise the majority of breast cancer BMET.

BMETs are uniquely increased (2-fold) in metastatic ER+ breast cancers as compared to metastases at other sites, where metastatic prevalence is either the same or reduced as compared to ER− tumors, and osteolysis is a bone- and tumor-specific event (e.g., primarily osteolytic in breast cancer *vs.* osteosclerotic in prostate cancer) known to be dependent on tumor-derived factors, such as PTHrP^[8–11]^. Thus, we posited that the apparent proclivity of bone-disseminated ER+ (*vs.* ER−) breast cancer cells to form clinically-evident osteolytic BMET could be attributable, at least in part, to pro-osteolytic effects of tumoral ERα signaling. The studies described here, which to our knowledge are the first to examine the E_2_ dose dependence of *in vivo* osteolytic ER+ BMET progression, support this postulate; over the range of E_2_ doses tested, while E_2_ effects on bone turnover or tumor cell seeding and proliferation in bone were constant, tumor-associated osteolysis and osteoclast formation at the bone/tumor interface in ER+ tumor-bearing mice increased in an E_2_ dose-dependent fashion, contrasting with well-described inhibitory effects of E_2_ on osteoclast formation in normal bone^[32,60]^. The additional finding of enhanced, E_2_ dose-dependent, ERα-regulated secretion of PTHrP, an osteolytic factor expressed in most clinical BMETs^[62,63]^, from BMET-derived ER+ breast cancer cells further supports this postulate and provides possible mechanistic insights for specific pathways downstream of tumoral ERα activation that may contribute to ER+ BMET-associated osteolysis. The enhanced secretion of PTHrP regulated by ERα from BMET-derived tumor cells, in particular, suggests: (1) ERα expression in ER+ cells metastatic to bone - rather than being just a biomarker for BMETs - may also be a potential molecular driver of osteolysis and metastatic progression in bone; and/or (2) either a subpopulation of highly PTHrP-expressing cells preferentially formed BMETs and/or the bone microenvironment altered the phenotype of bone-disseminated tumor cells to favor PTHrP-mediated osteolysis. Either of these possibilities is consistent with clinical observations that PTHrP-positivity in breast cancer is greater in BMET than in other metastatic sites or in primary tumors^[62]^, a finding also verified in pre-clinical murine studies documenting greater PTHrP expression in human breast cancer cells spontaneously forming metastases in bone *vs.* other sites^[73]^. The possible mechanistic importance of tumoral PTHrP secretion in promoting tumor-associated osteolysis and, in turn, osteolytic BMET progression, has already been established in one commonly studied pre-clinical ER− human BMET model, where osteolytic BMET progression does not occur in the absence of tumoral PTHrP bioactivity^[8,42]^. Also of particular relevance to the current studies, while E_2_-regulation of PTHrP expression in ER+ MCF7 cells has not, to our knowledge, been examined by laboratories other than our own^[74]^, overexpression of PTHrP by stable transfection in MCF-7 cells has been demonstrated to increase osteolysis specifically, in concert with a significant increase in osteolytic BMET progression (as compared to wild-type cells)^[26]^. Thus, existing evidence supports the postulate that enhanced secretion of PTHrP mediated by ERα in ER+ tumor cells disseminated to bone, as documented here, may be one specific pathway driving E_2_ dose-dependent tumor osteolysis and osteolytic ER+ BMET progression documented *in vivo*.

Clearly, though, these studies have limitations. Indeed, while a bone-specific hypothesis for tumoral ERα signaling driving BMET progression via mediation of tumor-associated osteolysis is straightforward, testing in pre-clinical models, where E_2_ supplementation is necessary to support robust progression of osteolytic BMET and a syngeneic mouse model is not available, is difficult since E_2_ has anabolic effects on the bone microenvironment and also clearly drives ER+ breast cancer cell proliferation, which is not unique to the bone microenvironment. Thus, while prior experiments utilizing E_2_-driven ER+ human breast cancer xenograft models and a single dose of E_2_ have demonstrated tamoxifen-inhibition of ER+ BMET following intracardiac tumor cell inoculation, or a role of zoledronic acid or tumor cell PREX1 expression in regulating dissemination of ER+ cells from primary orthotopic tumors ultimately home to bone^[18,21,27]^, none have been able to elucidate the relative importance of bone *vs.* tumor effects of E_2_, or other agents with dual bone *vs.* tumor effects, such as zoledronic acid. In the experiments described here, which are the first, to our knowledge, to test E_2_ dose dependency in an ER+ BMET model, the constancy across doses of E_2_-driven bone anabolism - an anticipated effect given E_2_’s known direct and/or indirect (via T and B lymphocytes, with only the latter being present in the model used here) stimulatory effects on osteoblasts and inhibition of myeloid-derived osteoclasts^[32,60]^ - could not account for the E_2_ dose-dependency of tumor-associated osteolysis. The osteolytic capacity of the ER+ tumors to overcome the marked increase in bone occurring in E_2_-treated mice, yielding osteolytic lesions similar in size and incidence to those reported in ER− models where anabolic increases in bone do not occur^[41]^, was also notable. However, the possibility that bone anabolism may have played a permissive, albeit constant, role in BMET progression in this ER+ model cannot be ruled out.

While the E_2_ dose-dependency of ER+ osteolytic BMET progression was not attributable to anabolic E_2_ bone effects given the constancy of this tumor microenvironment effect across doses, E_2_-driven bone anabolism clearly had independent pro-metastatic effects as well. Larger osteolytic lesion sizes in young (*vs.* mature) mice treated with the same E_2_ dose appeared attributable to greater E_2_-mediated anabolism in young mice since tumor cell dissemination and proliferation were otherwise the same. Increased osteolytic BMET lesion size in E_2_-treated (*vs.* control) mice inoculated with ER− breast cancer cells further confirmed a role of anabolic bone microenvironmental effects of E_2_ in driving osteolytic breast cancer BMET progression, independent of tumoral ER signaling, consistent with previous similar reports in ER− BMET models^[35,36]^. Because these experiments provide the first evidence, to our knowledge, that bone anabolic effects of E_2_ promote ER+ BMET progression subsequent to tumor cell dissemination to bone (as bone seeding was E_2_- and age-independent), this finding may have clinical implications when estrogens and/or other anabolic agents are used to treat osteoporosis in post-menopausal women^[75]^, an age where breast cancer incidence is the highest^[76]^ and silent bone micrometastases may already be present prior to a ER+ breast cancer diagnosis^[77–80]^. However, additional studies are required to explore this more specifically for both ER+ and ER− BMET, as, for example, studies evaluating anabolic effects of parathyroid hormone (PTH) on ER− BMET progression have yielded mixed results to date^[28,81,82]^. Additionally, it should be noted that the absence of an E_2_ effect on ER+ tumor cell dissemination to bone confirms previous reports^[20,27]^ and is consistent with the clinical observation of similar incidences of bone micrometastases in clinical series of patients with ER+ or ER− breast cancers^[77–80]^.

Lastly, the study of only a single ER+ breast cancer cell line in these pre-clinical experiments is a limitation. However, it should be noted that studies using breast cancer cells derived from a single ER− cell line (MDAMB-231), which shares fewer attributes with clinical breast tumors than the MCF-7 cells used here^[83]^, account for a large portion of pre-clinical breast cancer BMET research, but have still yielded important clinical insights, including the now standard therapeutic use of bisphosphonates for BMET^[84]^. Because of the reported low take-rates and rare formation of BMETs by ER+ patient derived xenografts (PDX)^[85–88]^, ER+ MCF-7 cells were initially chosen for these studies given their well-described ability to form osteolytic BMETs in E_2_-supplemented mice^[20–26]^. In addition, inoculation of other commonly used ER+ cell lines known to disseminate to bone (T47D and ZR-75-1)^[18,89]^ did not result in osteolytic BMET formation, with or without E_2_ supplementation (data not shown). However, this difference in osteolytic BMET potential between ER+ tumor cells provides evidence that the pro-osteolytic effects of E_2_ signaling in bone-disseminated ER+ breast cancer cells are likely also interdependent on other cellular transformations and signaling pathways present in ER+ tumor cells within the bone microenvironment - a postulate that awaits further testing.

In conclusion, while the study of ER+ breast cancer BMETs is complicated by the duality of ERα signaling effects in bone *vs.* bone-disseminated ER+ tumor cells, the experiments reported here, by taking advantage of differential dose-dependent effects of E_2_ on bone *vs.* ER+ tumor-associated osteolysis, suggest that ER+ osteolytic BMET progression may be specifically promoted by tumoral ERα signaling via the induction of osteolysis. Thus, additional bone-specific molecular targets downstream of ERα, in addition to those that drive proliferation, may complement existing therapeutics for the treatment of osteolytic ER+ BMETs, particularly for HT-resistant metastatic ER+ breast cancer, while potentially providing a mechanistic basis for the long-standing clinical observation of the association of tumoral ERα expression with breast cancer metastatic risk specific to bone.

## Supplementary Material

Supplementary Figure 1

Supplementary Figure 2

## Figures and Tables

**Table 1.** E_2_ effects on bone parameters in young and mature tumor-naive mice(mean ± SEM)^a^

			Young (4-week-old)				Mature (15-week-old)			Young *vs.* Mature	
	Mean (SEM)				*P*-values		Mean (SEM)		*P*-values	*P*-values	
	Control	E_2_, 0.05 mg	E_2_, 0.72 mg	Control *vs.* E_2_ 0.05 mg	Control *vs.* E_2_ 0.72 mg	E_2_ 0.05 mg *vs.* 0.72 mg	Control	E_2_, 0.72 mg	Control *vs.* E_2_ 0.72 mg	Control, Young *vs.* Mature	E_2_ 0.72 mg, Young *vs.* Mature
**Proximal tibiae bone density and volume** (6 weeks post-pellet)											
aBMD (mg/cm^2^)	89.3 (2.2)	151.1 (2.9)	143.9 (6.4)	< 0.0001	< 0.0001	n.s.	99.4 (4.8)	134.3 (4.5)	< 0.0001	n.s.	n.s.
BV/TV (%)	12.5 (2.0)	82.7 (2.7)	91.1 (2.4)	< 0.0001	< 0.0001	n.s.	10.1 (0.9)	59.5 (3.4)	< 0.0001	n.s.	<0.0001
**Bone turnover markers** (2 weeks post-pellet; relative to mature control)											
P1NP	1.8 (0.2)	2.4 (0.2)	2.5 (0.3)	0.0401	0.0197	n.s.	1.0 (0.1)	0.8 (0.1)	n.s.	0.0064	< 0.0001
CTX-1	1.43 (0.1)	1.9 (0.1)	1.5 (0.1)	0.0142	n.s.	n.s.	1.0 (0.2)	1.5 (0.2)	0.0066	0.0166	n.s.
**Bone cells** (2 weeks post-pellet)											
N.Ob/BS (mm)	33.1 (0.7)	36.1 (2.1)	39.18 (4.3)	n.s.	n.s.	n.s.	27.1 (6.6)	31.4 (4.2)	n.s.	n.s.	n.s.
N.Ob/mm^2^	393.4 (63.0)	765.8 (86.3)	849.2 (116.1)	0.0051	0.0011	n.s.	240.0 (70.61)	528.3 (5.8)	n.s.	n.s.	0.0278
N.Oc/BS (mm)	8.8 (1.1)	9.5 (0.6)	9.3 (0.4)	n.s.	n.s.	n.s.	7.6 (2.4)	8.3 (1.5)	n.s.	n.s.	n.s.
N.Oc/mm^2^	112.9 (22.7)	202.0 (22.9)	217.4 (14.6)	0.0237	0.0097	n.s.	64.7 (22.0)	170.1 (47.2)	0.0263	n.s.	n.s.

a *P*-values determined by1-way ANOVA with Fisher’s LSD test. N.Ob/BS (mm): Osteoblast number per bone surface; N.Ob/mm^2^: number of osteoblasts lining trabecular bone per tissue area; N.Oc/BS (mm): osteoclast number per bone surface; N.Oc/mm^2^: number of osteoclasts lining trabecular bone per tissue area; aBMD: areal bone mineral density; BV/TV: bone volume/total volume; n.s.: not significant.
