# Supplementary material for: Osteolytic effects of tumoral estrogen signaling in an estrogen receptor-positive breast cancer bone metastasis model
Source: J Cancer Metastasis Treat. Author manuscript; Available in PMC 2021 Nov 16. (PMC8594878; doi:10.20517/2394-4722.2021.27)
Supplement: Supplementary Figure 1 [file NIHMS1747926-supplement-Supplementary_Figure_1.pdf]

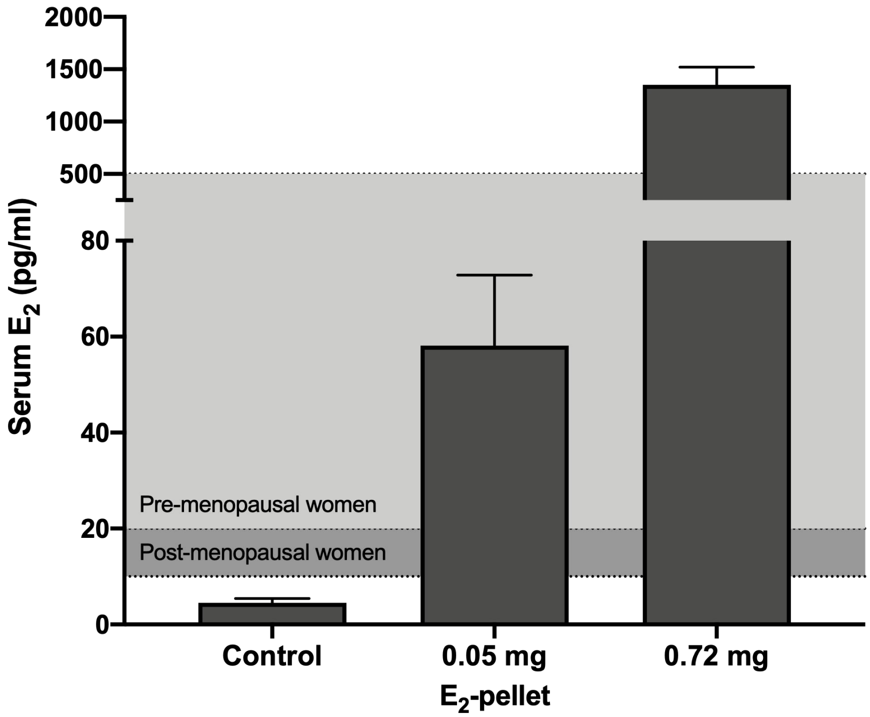

Supplemental Figure 1. Circulating E_2 levels in E_2-supplemented mice. Serum E_2 levels in naïve mice vs E_2 (0.72 mg 60-day pellet)-supplemented mice, 14 days post placement of pellets at 4 weeks of age. Assay limit of detection = 0.01 nM. Typical E_2 levels in pre-menopausal (20-500 pg/ml) and post-menopausal (10-20 pg/ml) women are indicated by shading for comparison[90].
