# Supplementary material for: Osteolytic effects of tumoral estrogen signaling in an estrogen receptor-positive breast cancer bone metastasis model
Source: J Cancer Metastasis Treat. Author manuscript; Available in PMC 2021 Nov 16. (PMC8594878; doi:10.20517/2394-4722.2021.27)
Supplement: Supplementary Figure 2 [file NIHMS1747926-supplement-Supplementary_Figure_2.pdf]

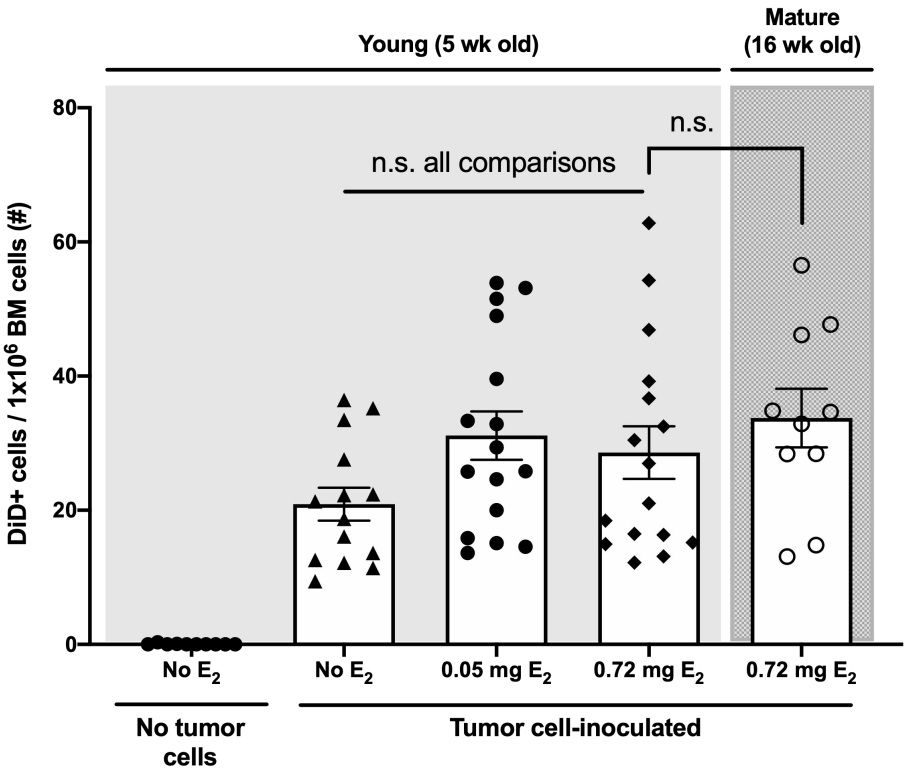

Supplemental Figure 2. E_2 effects on ER+ tumor cell seeding of bone. Quantification of tumor cells detected in proximal tibiae of E_2-supplemented mice (young or mature, as indicated) vs. mice with no E_2 supplementation, inoculated with DiD-labeled ER+ tumor cells. There were no significant differences (n.s.) in DiD+ tumor cell number in young mice with or without E_2, between E_2 doses (0.05 vs. 0.72 mg) in young mice, or between young and mature mice treated with 0.72 mg E_2, as tested by one-way ANOVA with Sidak's post-test (n=10-16/group). DiD+ cells were not detected in naïve mice lacking E_2-supplementation and tumor inoculation (n=10)
